# Water Deficit Affects the Growth and Leaf Metabolite Composition of Young Loquat Plants

**DOI:** 10.3390/plants9020274

**Published:** 2020-02-19

**Authors:** Giovanni Gugliuzza, Giuseppe Talluto, Federico Martinelli, Vittorio Farina, Riccardo Lo Bianco

**Affiliations:** 1 CREA—Research Centre for Plant Protection and Certification, SS 113 Km 245.500, 90011 Bagheria, Italy; giovanni.gugliuzza@crea.gov.it (G.G.); agronomo.talluto@libero.it (G.T.); 2 Department of Biology, University of Florence, via Madonna del Piano 6, 50019 Sesto Fiorentino, Italy; federico.martinelli@unifi.it; 3 Department of Agricultural, Food and Forest Sciences, University of Palermo, Viale delle Scienze, Ed. 4, 90128 Palermo, Italy; vittorio.farina@unipa.it

**Keywords:** drought, dry weight, *Eriobotrya japonica*, photosynthesis, sorbitol, stem water potential

## Abstract

Water scarcity in the Mediterranean area is very common and understanding responses to drought is important for loquat management and production. The objective of this study was to evaluate the effect of drought on the growth and metabolism of loquat. Ninety two-year-old plants of ‘Marchetto’ loquat grafted on quince were grown in the greenhouse in 12-liter pots and three irrigation regimes were imposed starting on 11 May and lasting until 27 July, 2013. One-third of the plants was irrigated with 100% of the water consumed (well watered, WW), a second group of plants was irrigated with 66% of the water supplied to the WW plants (mild drought, MD), and a third group was irrigated with 33% of the water supplied to the WW plants (severe drought, SD). Minimum water potential levels of −2.0 MPa were recorded in SD plants at the end of May. Photosynthetic rates were reduced according to water supply (WW > MD > SD), especially during the morning hours. By the end of the trial, severe drought reduced all growth parameters and particularly leaf growth. Drought induced early accumulation of sorbitol in leaves, whereas other carbohydrates were not affected. Of over 100 leaf metabolites investigated, 9 (squalene, pelargonic acid, glucose-1-phosphate, palatinol, capric acid, aconitic acid, xylitol, lauric acid, and alanine) were found to be useful to discriminate between the three irrigation groups, suggesting their involvement in loquat metabolism under drought conditions. Loquat behaved as a moderately drought-tolerant species (limited stem water potential and growth reductions) and the accumulation of sorbitol in favor of sucrose in mildly-stressed plants may be considered an early protective mechanism against leaf dehydration and a potential biochemical marker for precise irrigation management.

## 1. Introduction

Loquat (*Eriobotrya japonica* Lindl.) is a subtropical evergreen tree native to southeastern China [1], but is easily cultivated in all areas of southern Mediterranean countries. Differently than most temperate fruit trees, loquat flowers in autumn, develops its fruits during winter, and matures them in early spring. This total lack of overlap between shoot and fruit growth allows loquat to generally escape water deficit during fruit development under standard Mediterranean conditions. As a result, summer deficit irrigation is today a successful practice used to anticipate flowering and fruit ripening without yield reductions [2,3,4,5].

Leaf area index is often reduced by drought, as the leaf blade is one of the most sensitive plant organs to dehydration [6,7,8,9,10]. Response to occasional drought events is more complex in trees than in annual crops and it does not necessarily result in the reduction of yield. Trees may indeed resist temporary water deficit, decreasing trunk, branch, or shoot growth, while maintaining plant productivity since fruits and seeds are generally stronger sinks than shoots under limited carbon assimilation [3,11,12].

Osmotic adjustment, i.e., the active accumulation of solutes within cells, is typically observed in plants under drought, salt, or temperature stresses and allows turgor or cell volume to be maintained to the level of non-stressed plants [13,14]. Studies with plants genetically modified to favor osmotic adjustment have shown that solute accumulation may result in improved tolerance to some dehydration stresses [15,16,17], indicating that osmotic adjustment is not due to just a passive solute accumulation during dehydration.

There is considerable interest in the types of solutes used for osmotic adjustment and in where and how their accumulation occurs and is regulated. During drought stress, organic compounds, such as polyols, sugars, proline, and glycine-betaine, comprise the bulk of solutes used for osmotic adjustment [18]. In Rosaceous tree fruits, such as peach (*Prunus persica* Batsch.) [19], apple (*Malus domestica* [L.] Borkh.) [20], and cherry (*Prunus cerasus* L. and *P. avium* X *pseudocerasus*) [21], osmotic adjustment during drought stress is facilitated mainly by sorbitol accumulation. Sorbitol accumulation in apple leaves may also represent a good biochemical marker of moderate drought stress and a useful parameter for developing precise irrigation schedules in apple orchards [22]. Water stress also induces sorbitol accumulation in loquat leaves [23]. Sorbitol or other polyols may function as compatible solutes in transgenic tobacco [24], *Plantago* spp. [25], *Hedera helix* (L.) [26], and members of the Oleaceae family [27,28]. Furthermore, polyols have several functions other than osmotic adjustment, such as translocation and storage of carbon, cryoprotection, prevention of activated oxygen species, boron transport, and energy delivery [29,30].

In addition to primary metabolites, plant secondary metabolites are also well known to be differentially expressed in response to abiotic stress and, in particular, oxidative stress induced by dehydration. Specifically, they are able to counteract redox state changes, providing plant stress resistance [31,32,33,34,35,36].

All these changes induced by drought may greatly affect loquat growth and yields. Hence, we conducted an experiment to investigate the effect of drought on the growth and metabolite changes of young loquat trees.

## 2. Results and Discussion

### 2.1. Water Relations, Gas Exchange, and Growth

**Figure 1:**
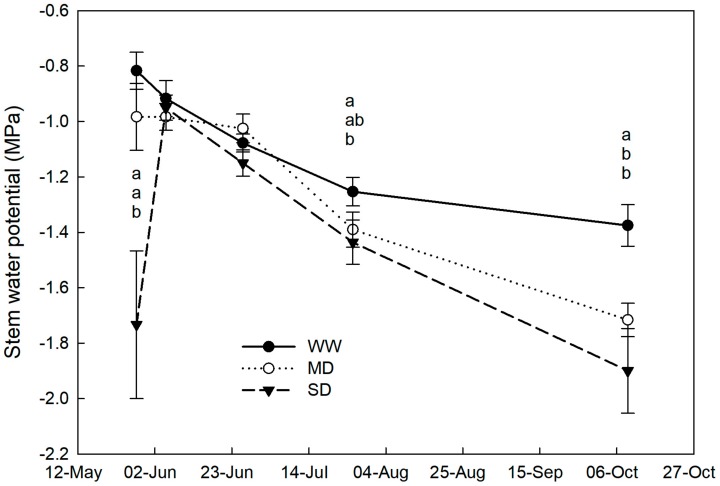
Stem water potential in loquat plants under well-watered conditions (WW), mild-drought (MD), and severe drought (SD) during the four months of the trial. Error bars represent standard errors of means. When present, different letters indicate significant differences among drought treatments for a specific date (Tukey’s multiple range test, *p* < 0.05).

In well watered (WW) plants, stem water potential (WPstem) exhibited a steady trend during the trial period and remained within −1.3 MPa (Figure 1). Also, the WPstem of plants under mild drought (MD) was similar to the WPstem of WW plants in the first part of the trial and significantly lower than the WPstem of WW plants only at the end of the trial. On the other hand, the WPstem of plants under severe drought (SD) was already significantly lower than the WPstem of WW plants after just 18 days from the beginning of drought and by the end of the drought period (end of July). The lowest WPstem levels of −2.0 MPa were reached by SD plants at the end of the trial (Figure 1).

Similar levels of water potential were observed in field-grown loquat trees under 55% [2], 50%, and 25% [4] irrigation deficit. Loquat plants of the present trial tended to resist drought by avoiding it as they were able to maintain acceptable hydration levels. A 2% (*p =* 0.045) and 3% (*p =* 0.021) unit DW (calculated as a percentage of TW) increase in MD and SD leaves, respectively, was detected only at the end of July (77 days after the beginning of drought conditions), suggesting some osmotic adjustment in the drought treatments compared to control plants.

**Figure 2:**
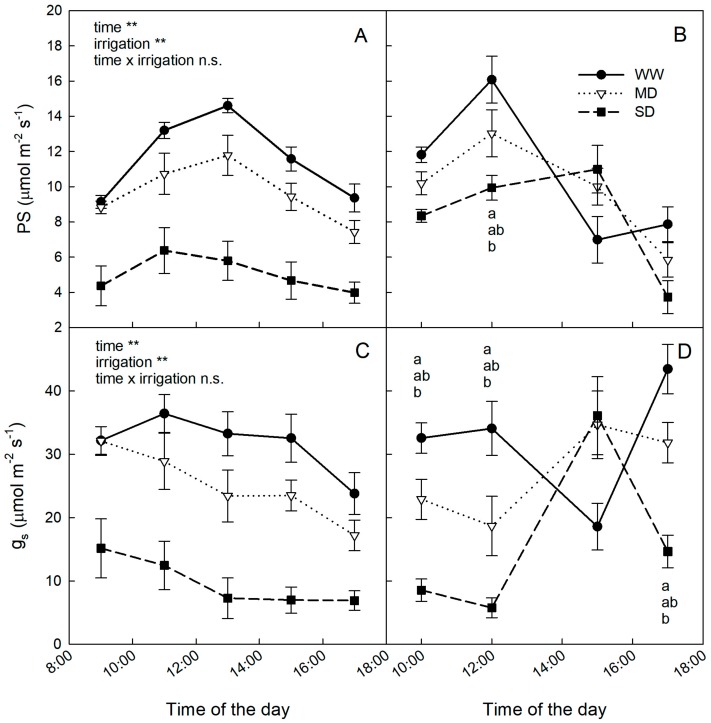
Daily trends of photosynthetic rate (PS) and stomatal conductance (g_s_) in loquat plants under well-watered conditions (WW), mild drought (MD) and severe drought (SD) on 29 May ((**A**) and (**C**)) and 26 July ((**B**) and (**D**)). Error bars represent standard errors of means. Significance level from analysis of variance: n.s., non-significant; **, significant at P < 0.001. When present, different letters indicate significant differences among irrigation treatments for a specific date (Tukey’s multiple range test, *p* < 0.05).

Photosynthetic rates were significantly reduced according to water supply (WW > MD > SD), especially in May when plants were not prepared to tolerate dehydration. At this time, the photosynthetic rate (PS) of SD plants was reduced by about 50% (Figure 2A).

At the end of July, PS was mainly reduced by drought at noon when atmospheric water demand was highest (Figure 2B). The latter may be explained by stomatal conductance (g_s_) reductions at midday (Figure 2C,D). Indeed, g_s_ followed daily trends similar to PS on both dates (Figure 2), indicating a stomatal limitation mechanism for photosynthesis.

**Figure 3:**
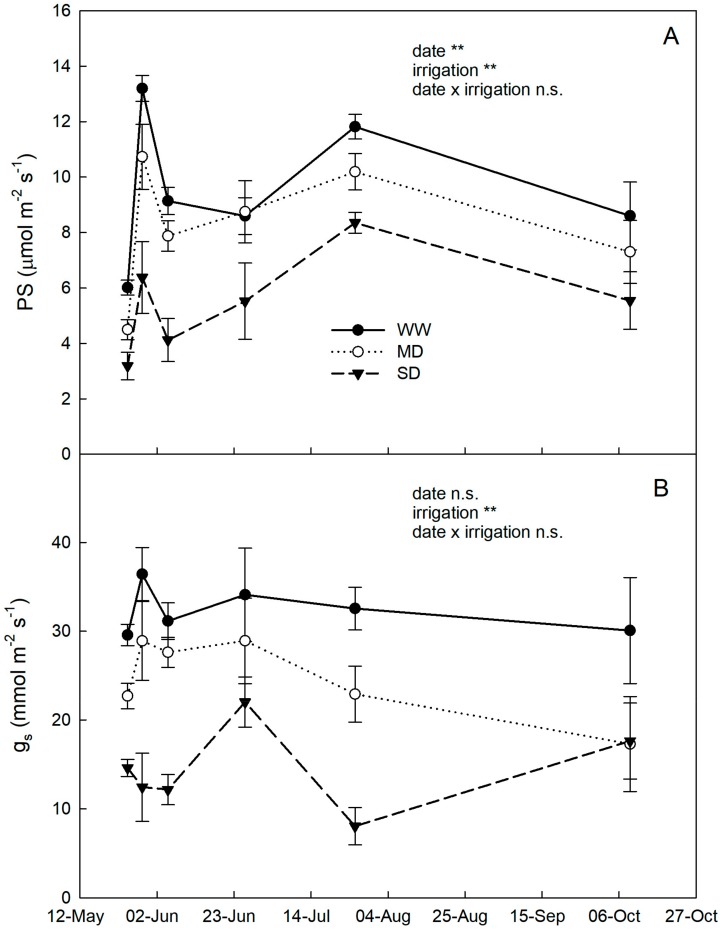
Photosynthetic rates (PS) (**A**) and stomatal conductance (g_s,_) (**B**) in loquat plants under well-watered conditions (WW), mild drought (MD) and severe drought (SD) during the four months of drought. Significance level from analysis of variance: n.s., non-significant; **, significant at *p* < 0.001. Error bars represent standard errors of means.

Photosynthesis and g_s_ across the entire trial followed similar trends in the three treatments and were significantly and consistently reduced according to water supply (WW>MD>SD) (Figure 3). Our findings agree with reductions in photosynthetic rates observed in loquat under water stress by Huajiang et al. [37] and Stellfeldt et al. [38].

By the end of the experiment, SD affected all growth parameters and particularly leaf growth, reducing total leaf dry weight (TLDW) by 35% and total leaf area (TLA) by 50%, and increasing specific leaf weight (SLW) by 28% (Table 1). On the other hand, MD did not affect stem diameter and SLW. The observed growth reductions agree with data reported by other investigators in various tree species [3,7,8,9] and also in loquat under salt stress [39]. No significant change in root/shoot ratios was detected (data not shown).

### 2.2. Soluble Carbohydrates

**Figure 4:**
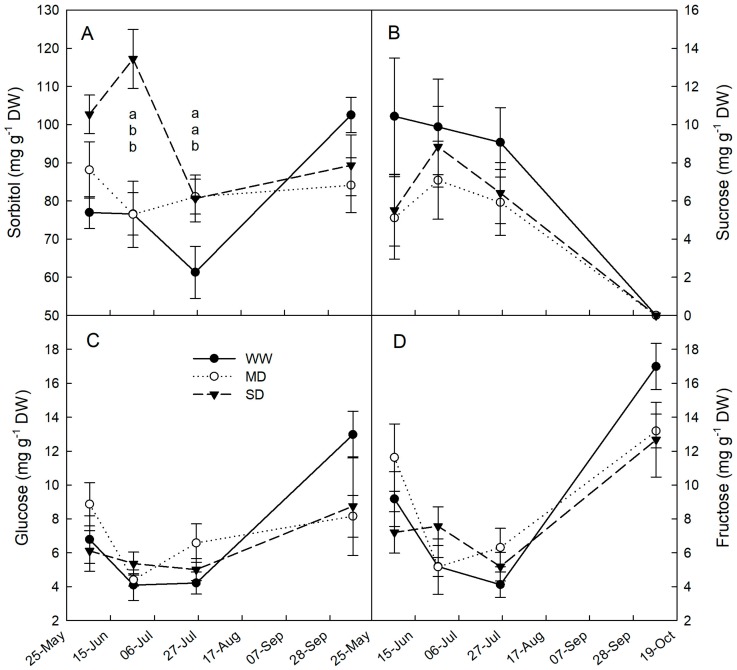
(**A**) Sorbitol, (**B**) sucrose, (**C**) glucose, and (**D**) fructose content in leaves of loquat plants under well-watered conditions (WW), mild drought (MD), and severe drought (SD) during the four months of drought. Error bars represent standard errors of means. When present, different letters indicate significant differences among drought treatments for a specific date (Tukey’s multiple range test, *p* < 0.05).

In SD plants, leaf sorbitol accumulation was observed by late June, after about a month of drought treatment, whereas in MD plants, sorbitol accumulation was detected only at the end of the drought period, about a month later (Figure 4A). Similarly, sorbitol accumulation was documented in the leaves and roots of young loquat trees under water stress [23]. Sorbitol also accumulated in mature leaves of peach under drought stress, contributing significantly to osmotic adjustment [19]. Also, mannitol accumulated under water deficit conditions and may function as a compatible solute in transgenic tobacco [24,40] and members of the Oleaceae family [27,28,41]. The leaf sorbitol of SD and MD plants was similar to that of WW plants after the long period of rewatering in the field (Figure 4A). No significant increase in sucrose, glucose, or fructose was detected in response to drought (Figure 4B–D).

Average sorbitol/sucrose ratios during the drought period were significantly higher in MD (13.5) and SD (14.2) compared to WW (7.4) leaves. This is a clear indication of carbon allocation shifts toward more osmotically-active compounds under drought conditions. Regardless of drought treatment, both glucose and fructose increased significantly in early October (Figure 4C,D) at the expenses of sucrose (Figure 4B). This may indicate a significant increase in leaf metabolism and respiration as the cooler season approaches, but it may also indicate a transitional accumulation of hexoses for the imminent synthesis of starch, as previously observed in water-stressed loquat leaves [23].

### 2.3. Metabolomic Analysis

**Figure 5:**
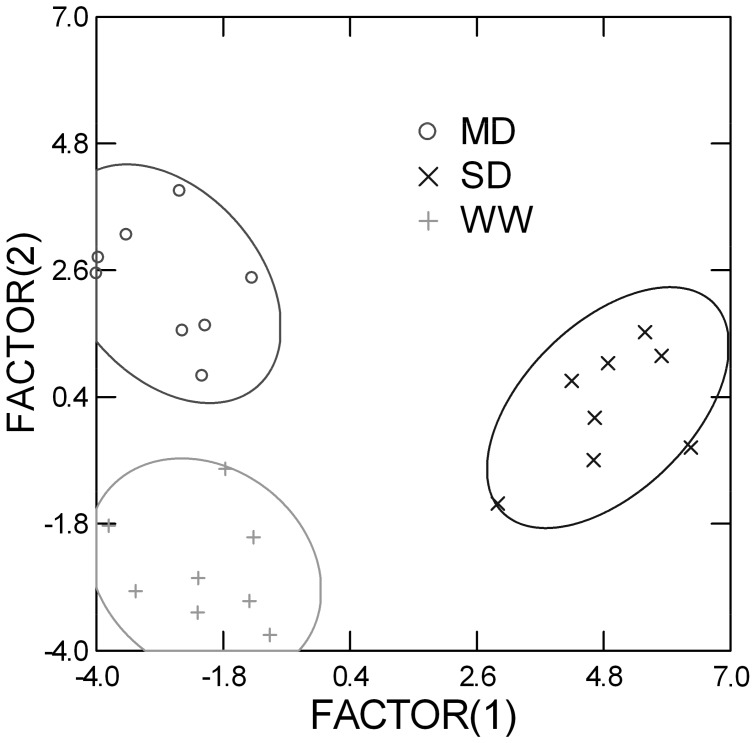
Canonical score plot from linear discriminant analysis of metabolites detected in leaves of loquat plants under well-watered conditions (WW), mild drought (MD) and severe drought (SD) during the four months of trial.

Metabolomic analysis determined the relative quantitative levels of more than 100 metabolites belonging to primary (carbohydrates, amino acid, fatty acids and others) and secondary metabolism among the three drought treatments (Table S1). LDA of all leaf metabolites was able to completely separate the three drought treatments (Figure 5). Specifically, the canonical score plot showed a smaller distance between WW and MD than between MD (or WW) and SD, suggesting that MD had a minor effect on plant metabolism compared to SD. In this case, the canonical discriminant functions after a forward step analysis included the following nine metabolites: squalene, pelargonic acid, glucose-1-phosphate, palatinol, capric acid, aconitic acid, xylitol, lauric acid, and alanine.

Squalene is a polyunsaturated triterpene widely produced by plants and particularly present in olives, wheat germ, and rice bran at relatively high concentrations [42]. It is also a major component of olive oil, reaching more than 90% of the secondary metabolite fraction [43]. This terpenoid has a well-known protective role against various oxidative stresses due to its chemo-protective role in skin cancer and aging [44]. Squalene is a precursor of sterols and is very important for virgin olive oil authentication [45], ranging between 800 to 900 and 6000 to 8000 mg kg^−1^ in different varieties [46,47,48]. Irrigation is one of the main agronomic practices affecting squalene levels of olive oils [49]. Interestingly, squalene was significantly higher in plants irrigated with wastewater than good quality water [46], with values reaching those expected for good quality olive oil [50]. This effect has been linked with the higher lectrical conductivity of treated wastewater irrigation as it has been observed previously for tocopherols [51]. A metabolomic analysis of olive fruit showed a positive effect of irrigation on squalene amounts [52]. An increase in squalene under well-watered conditions has also been observed previously in olive fruit of Leccino [53], Barnea, and Souri [54] cultivars. Another possible explanation is that this enhanced amount might be due to increased squalene biosynthesis or caused by the reduced competition of metabolites sharing the same precursor (3S)-2,3-oxidosqualene [55]. An analysis of squalene content during ripening highlighted that, regardless of irrigation, this compound is reduced when the ripening of the olive fruit begins [56]. As squalene is a carotenoid metabolite, the increase in response to irrigation could be expected, considering that it is one of the hydrocarbon compounds that contributes to transport energy and that it also protects the oxidation of chlorophyll [52,57]. Water supply should enhance photosynthesis in leaves and, consequently, enhance the production of compounds, like squalene, protecting chlorophyll from oxidative stresses [58]. It is worth noting that squalene content is very variable and affected mainly by genetic and climate factors [59]. Taking together these previous findings and our results, we can conclude that data from the present study also confirm the link between squalene and responses to drought in loquat.

As for carbohydrates, water stress reduced the amounts of glucose, fructose, and sucrose in leaves of groundnut (*Arachis hypogaea* L.) [60]. This was linked with a reduction of chlorophyll content and a consequent reduction in photosynthesis. In olives, for example, glucose (as well as other simple carbohydrates) was higher in leaves of irrigated trees compared to drought-stressed trees, although this type of response seems to be cultivar-specific [41,61]. Indeed, it is worth mentioning that contrasting evidence has been also published. For example, in leaves of the olive cultivar Picual, glucose content increased in response to water stress [62]. These contrasting results may be attributed to the degree of water deficit both in terms of time and intensity, with severe stress conditions inducing a general decline of total carbohydrate production.

In this study, capric acid was one of the metabolites that contributed significantly to discriminating between drought-stressed and well-irrigated plants. Capric acid, also known as decanoic acid, is a saturated fatty acid that belongs to the category of short-chain fatty acids [63]. Their role, together with abscisic acid, has been related to the control of stomatal closure in response to water stress [64]. Capric acid is one of the straight-chain saturated fatty acids (C6-C11) that increased in leaves of common bean and *Hordeum vulgare* (L.) under water deficit [58]. In particular, capric acid was one of the most effective fatty acids at provoking stomatal closure and preventing opening with a consequent substantial cell-membrane leakage in *Commelina communis* (L.) [64]. Indeed, it has been speculated that the carbon-chain length may be an essential factor in determining the occurrence of membrane leakage and damage of internal cell structures.

Another metabolite found to be significantly modulated by drought stress in loquat was aconitic acid. It is one of the key organic acids and intermediate of the tricarboxylic acid (TCA) cycle, which plays an important role in ATP biosynthesis through energy metabolism in plants [65], and it is modulated by abscisic acid (ABA), a key player in plant abiotic stress responses [66].

Xylitol has been found in low amounts in plums, strawberries, pumpkin, and cauliflower and it has been shown to be affected by water deficit in grapevines, where it is involved in mechanisms of stress tolerance [67]. Together with other well-known polyols, Conde et al. [67] concluded that the accumulation of xylitol might be effective in maintaining cell and tissue homeostasis by acting as an osmoprotectant. Our work was the first to find this sugar alcohol in loquat leaves and seems to confirm Conde et al.’s hypothesis.

Lauric acid is a dodecanoic acid, a saturated fatty acid having similar properties of medium-chain fatty acids. It is present in several plants, such as babassu palm (*Attalea speciosa* Mart.), cohune palm (*Attalea cohune* Mart.), peach palm, date palm, plum, and watermelon [68]. Although the role of lauric acid in response to water stress has to be still elucidated, it is interesting that the presence of lauric acid in the in vitro culture medium was found to promote resistance to drought stress at the pre-acclimatization stage in *Syagrus coronata* (Mart.) [69]. These plants modified their metabolic rates during water deficit by maintaining leaf water concentrations, modulation of stomatal closure, and dissipation of non-photochemical energy. The same plants increased photosynthesis during the recovery stage. All of these findings let us speculate that lauric acid might have a role similar to capric acid and ABA to improve stomatal sensitivity and enhance drought resistance in loquat leaves.

The canonical discriminant analysis found that alanine was associated with drought response in loquat. This amino acid has been previously found to be affected by drought in *Thymus vulgaris* (L.) and *T. kotschyanus* (L.) and linked to relative water content (RWC) [70]. In both species, alanine and choline were similarly increased under drought stress. In particular, a moderate stress increased the alanine amount, while a severe stress showed no significant changes [70]. Alanine, together with other amino acids (i.e., valine, threonine, and glycine), is known to work as an osmoprotectant in higher plants, including grapevine [71,72,73]. Previous works reported that drought stress enhances alanine amount through the biosynthesis of pyruvate in *Brassica napus* (L.) [74]. A post-transcriptional omic analysis in tea plant (*Camellia sinensis* L.) highlighted that some miRNAs, whose targets are involved in alanine metabolism, were differentially regulated by drought, implying that alanine-related pathways are affected by water deficit and that these changes are modulated by miRNAs [75]. Amounts of amino acids were higher in leaves of water-stressed satsuma orange leaves [76]. On the other hand, a reduction of alanine and other amino acids was found in the leaves of soybean genotypes, which are known to be relatively sensitive to water deficit [77]. In maize leaves, alanine and other amino acids accumulated during drought, although the response of these compounds varied depending on the cultivar, organ, and developmental stage [78]. In two *Phyllanthus* species, drought stress increased the levels of several organic acids and amino acids, including alanine, which worked as an osmoprotectant [79].

## 3. Materials and Methods

### 3.1. Plant Material and Experimental Design

The experiment was carried out in a greenhouse located at the Research Centre for Plant Protection and Certification in Bagheria, Sicily, transmitting about 70% of the integrated daily solar radiation and with temperatures ranging from 10 to 35 °C in May, from 12 to 37 °C in June, and from 15 to 38 °C in July, 2013; average daily relative humidity was 45%, 47%, and 44% in May, June, and July, respectively. Ninety two-year-old and uniform in size loquat plants (cultivar Marchetto grafted on quince rootstock, *Cydonia oblonga* Mill.) were grown in 12-liter plastic containers. The soil medium was made of sand (35%), peat moss (50%), and perlite (15%), with 40% water content at field capacity.

From 11 May until 27 July, 2013 plants were supplied with three irrigation volumes. One-third of the plants was irrigated to the point of runoff (WW) three times a week to replace 100% of water lost to evapotranspiration (ET). The daily ET of WW plants was determined by weighing each pot to the nearest gram before every irrigation. On the same days, the remaining plants were supplied with irrigation volumes equal to 66% (MD) and 33% of ET (SD). In MD and SD plants, water was supplied to plastic saucers beneath the pots to reach the growing roots and minimize surface evaporation. All plants were supplied with slow-release fertilizer in the soil medium before the drought trial started. Plants were divided into three randomized blocks, with 10 plants per treatment in each block. In August, plants were moved outside the greenhouse and watered regularly.

### 3.2. Growth, Water Relations, and Gas Exchange

All plants showed active growth during the entire trial and height, basal diameter, total leaf area (TLA), total leaf dry weight (TLDW), and specific leaf weight (SLW) were determined after nine weeks of drought treatment.

Stem water potential (WPstem) was measured during the drought period and after a period of rewatering to full ET rates with a Scholander pressure chamber on 10 plants per treatment. Leaf disks from the same plants and on the same dates as the WPstem measurements were collected to determine fresh (FW) and dry (DW) weights after oven drying at 60 °C to a constant weight. After measuring FW, leaf disks were soaked in deionized water for 4 h and turgid weight (TW) was recorded with a precision scale. Leaf relative water content (RWC) wad calculated as (TW − FW)/(TW − DW).

At the beginning (May 29) and at the end (July 26) of the drought period, a LI-COR 6400 portable system (LI-COR, Lincoln, NE, USA) was used to measure gas exchange on one leaf from six plants per treatment throughout the day.

### 3.3. Soluble Carbohydrates

Disks from the same leaves as those used for RWC determinations (mature, one-year-old leaves) were collected and stored at −40 °C for later analysis of soluble carbohydrates. About 0.1 g of leaf blade was cut in small pieces, transferred into 1.5-mL Eppendorf tubes, and finely ground with a V-shaped pestle under liquid nitrogen. Powdered samples were weighed and extracted with 1 mL of 80% (v:v) methanol/water solution. The suspension was vortexed for 1 min and centrifuged for 5 min at 3000× *g*. The supernatant was stored at −40 °C for later determination of glucose, fructose, sucrose, and sorbitol content by liquid chromatography/mass spectrometry (LC/MS) using Thermo TSQ Quantum Access equipment and a Hypercarb PGC (100 mm × 2.1 mm, 5 µm) column (Thermo Fisher Scientific Inc., Waltham, MA, USA). Extraction and quantification procedures were performed by the Centro Grandi Apparecchiature (UniNetLab), University of Palermo. Glucose, fructose, sucrose, and sorbitol standards were used to obtain calibration curves (R^2^ > 0.9). All reagents were purchased from Sigma Aldrich (St. Louis, MO, USA). Carbohydrate contents were expressed in mg per g of DW.

### 3.4. Metabolomic Analysis

Six leaf samples (mature, one-year-old leaves) from three plants under each of the three drought treatments were collected for the metabolomic analysis. For each sample, 20 mg of ground tissue was collected and 2 mL of prechilled extraction solvent (MetOH:CHCl3:1:1) (v/v) was added and stirred at 4 °C for 5 min. After vortexing, the extract was centrifuged at 6100 g for 2 min and aliquots of 20 μL of supernatant were completely dried. Derivatization of samples was performed using methoxyamine and N-methyl-N-(trimethylsilyl)trifluoroacetamide and samples were subsequently analyzed using the Agilent 6890GC–quadrupole mass spectrometer (MS). The temperature of the Agilent oven was increased by 10 °C every min starting at 60 °C (1 min initial time) to 325 °C (10 min final time), resulting in a 37.5 min run time with cooling down to 60 °C. A 10-μL syringe was used to inject 1 μL into the Agilent split/splitless injector at 250 °C with four sample pumps. One pre-injection wash and two post-injection washes were performed. No viscosity delay or dwell time was applied, using a fast plunger speed. Samples were introduced in both splitless and split conditions. A helium purge flow of 10.5 mL min^−1^ was used (splitless conditions, 1 min at 8.2 psi). A constant helium flow rate of 1 mL min-1 was used as the carrier gas. The quadrupole MS was switched on after a 5.9 min solvent delay time, scanning from 50 to 600 u. The source temperature was 230 °C and the quadrupole temperature was 150 °C. Prior to acquisition, the MSD was autotuned using FC43 following the instrument calibration procedure. When using split injections, parameters used were identical to those given above but with a split ratio of 1:10 and a split flow rate of 10.3 mL min^−1^. When data were acquired, missing values in the data matrix were replaced with “XX”. Compounds that were not detected in at least 10% of the sample within a class were discarded. All detected peaks were controlled for false positive and false negative assignments. The metabolite identification was conducted using the Agilent Fiehn GC/MS Metabolomics RTL Library. Data were analyzed using Agilent Mass Profiler Professional Software using default parameters for noise reduction, normalization, mass spectral, and compound identification. Internal standards were injected at the moment of the analysis for each sample. Internal standards C08-C30 fatty acid methyl esters were added and the sample was derivatized by methoxyamine hydrochloride in pyridine and subsequently by N-methyl-N-trimethylsilyltrifluoroacetamide for trimethylsilylation of acidic protons. The ‘internal standard’ addition within the BinBase name clarifies if a specific chemical has been added into the extraction solvent as an internal standard. These internal standards serve as retention time alignment markers for quality control purposes and for quantification corrections. The following equation was used to normalize peak height for metabolite i of sample j and internal standard k:

metabolite ij, normalized = metabolite ij, raw/istd k × concentration istd k.

Relative concentrations were determined by ratios of the peak area between each identified metabolite and the eight internal standards (mm^2^ mm^−2^). Extraction and quantification procedures were performed by the West Coast Metabolomics Center, University of California, Davis, CA, USA.

### 3.5. Data Analysis

Analysis of variance was used to analyze growth, WPstem, RWC, and carbohydrate data and was followed by Tukey’s multiple comparison test to detect significantly different means. Metabolomic data were analyzed using linear discriminant analysis with a forward stepwise procedure. All tests were carried out using procedures of the Systat software package (Systat Software Inc., Richmond, CA, USA).

## 4. Conclusions

Overall, the young loquat plants monitored in this study were moderately drought tolerant (limited WPstem and growth reductions) and, in most cases, severe drought conditions were needed to detect significant effects. Indeed, in terms of growth, gas exchange, and metabolism, plants under mild drought conditions were generally more similar to well-watered plants than to plants under severe drought condition. This may be in part justified by specific modifications of carbohydrate allocation patterns toward osmotically more active forms. In particular, the accumulation of sorbitol in favor of sucrose that also occurred in mildly-stressed plants may be identified as a protective mechanism against leaf dehydration and related water stress effects already at early stages of water deficit. For this reason, sorbitol may be considered a good biochemical marker of water deficit, showing a good potential for precise irrigation management in loquat. Under more severe drought conditions (i.e., SD), sorbitol accumulation was even more evident, but unable to protect loquat plants from leaf dehydration and related effects (i.e., reductions in growth and photosynthesis). Other metabolic changes typical of water-stressed plants were detected in loquat leaves, indicating the mediation mechanisms between leaf dehydration sensing and final growth outcomes.

## Figures and Tables

**Table 1:** Final height, main stem diameter, total leaf dry weight (TLDW), total leaf area (TLA), and specific leaf weight (SLW) in loquat plants under well-watered conditions (WW), mild drought (MD) and severe drought (SD) Within each column, different letters indicate significantly different means by Tukey’s multiple range test (*p* < 0.05).

Drought Treatment	Height (cm)	Diameter (cm)	TLDW (kg)	TLA (m^2^)	SLW (cm^2^)
WW	113 a	2.00 a	0.129 a	0.74 a	0.174 b
MD	102 b	2.00 a	0.099 b	0.56 b	0.177 b
SD	101 b	1.81 b	0.081 b	0.35 c	0.231 a

## Supplementary Materials

The following are available online at https://www.mdpi.com/2223-7747/9/2/274/s1, Table S1: Leaf metabolites analyzed using GC-MS-TOF in loquat plants under well-watered conditions (WW), mild drought (MD) and severe drought (SD) during the 9-weeks of drought. Amounts for each metabolite are relative concentrations determined comparing sample peak areas (mm^2^) with peak areas of injected standards (n = 8).

## Abbreviations

ABA, abscisic acid; DW, dry weight; FAME, fatty acid methyl ester; FW, fresh weight; g_s_, stomatal conductance; LDA, linear discriminant analysis; MD, mild drought; PS, photosynthetic rate; RWC, relative water content; SD, severe drought; SLW, specific leaf weight; TLA, total leaf area; TLDW, total leaf dry weight; TW, weight at full turgor; WPstem, stem water potential; WW, well watered.
