# Supplementary material for: Water Deficit Affects the Growth and Leaf Metabolite Composition of Young Loquat Plants
Source: Plants (Basel). 2020 Feb 19;9(2):274. doi: 10.3390/plants9020274 (PMC7076381; doi:10.3390/plants9020274)
Supplement: Supplementary file 1 [file plants-09-00274-s001.pdf]

Supplementary Materials

Table S1. Leaf metabolites analyzed using GC-MS-TOF in loquat plants under well-watered conditions (WW), mild drought (MD) and severe drought (SD) during the 9-weeks of drought. Amounts for each metabolite are relative concentrations determined as a ratio between sample peak areas (mm^2) with peak areas of injected standards (n = 8) (mm^2).

Metabolite	WW	MD	SD
quinic acid	391004	392916	367226
malic acid	56552	47165	45988
stearic acid	53842	52195	50913
hydroxylamine	40193	36173	42979
shikimic acid	23683	17242	16542
citric acid	21643	25430	17085
chlorogenic acid	11763	3713	4238
galactinol	11747	16257	16986
beta-gentiobiose	9389	7935	8317
epicatechin	9076	5118	3114
xylitol	8107	8097	11852
palmitic acid	7548	7181	7084
myo-inositol	6356	7053	6256
6-deoxyglucose	6232	5380	7027
N-acetylmannosamine	4871	4157	4286
glycerol-3-galactoside	4084	5128	4077
gluconic acid	3927	3073	3095
beta-sitosterol	3589	3781	4540
hydroxycarbamate	2951	2405	3237
alanine	2930	2196	3521
ethanolamine	2847	2510	3239
xylose	2266	2502	2166
glyceric acid	2126	1602	1520
glycerol	1940	1881	1954
ribitol	1845	2016	2555
methyltetrahydrophenanthrenone	1632	1634	1690
lauric acid	1474	1584	1737
glucoheptulose	1411	1470	1487
valine	1344	423	1980
3,4-dihydroxybenzoic acid	1307	1122	865
glutamic acid	1190	982	1325
pelargonic acid	1164	1096	1551
glucose-1-phosphate	1123	924	1022
squalene	1119	1142	2725

tocopherol alpha-	1086	622	746
lactic acid	1024	817	1227
beta-mannosylglycerate	980	774	918
oxoproline	934	753	1035
phosphate	922	804	1052
succinic acid	870	795	615
trehalose	843	1022	923
arachidic acid	797	837	891
lyxitol	784	824	983
maleic acid	781	556	582
palatinitol	776	1581	677
urea	760	631	996
isochlorogenic acid	742	411	384
sophorose	687	549	674
heptadecanoic acid	677	684	689
threonic acid	658	503	594
tryptophan	641	328	732
isoleucine	613	187	968
ribose	611	676	737
leucrose	598	483	489
6-deoxyglucitol	566	751	904
phytol	539	464	585
aspartic acid	489	419	754
hexonic acid	487	521	563
fumaric acid	484	406	487
benzoic acid	468	505	555
erythritol	466	462	553
gluconic acid lactone	429	426	430
aconitic acid	427	220	345
maltitol	384	439	421
phenol	371	327	451
saccharic acid	347	405	357
glycolic acid	290	231	262
proline	290	179	253
serine	273	124	243
lactose	272	229	311
levoglucosan	269	313	365
pentitol	258	275	255
caprylic acid	256	242	303
3,4-dihydroxycinnamic acid	246	168	177

linolenic acid	238	270	187
xylonic acid	237	187	136
butyrolactam	232	224	268
adenine	230	268	230
4-aminobutyric acid	224	224	276
threitol	209	205	244
2-ketoglucose dimethylacetal	209	166	149
4-hydroxycinnamic acid	208	206	158
mucic acid	193	216	168
fucose	183	198	174
ribonic acid	179	183	168
phenylalanine	177	175	183
octadecanol	172	175	166
2-hydroxyglutaric acid	169	144	149
raffinose	159	126	213
1-hexadecanol	148	186	182
isothreonic acid	143	142	172
alpha-ketoglutarate	142	128	108
linoleic acid	115	138	149
glycine	113	91	92
capric acid	109	142	114
dodecanoic acid, isopropanol ester	89	114	111
oleic acid	85	84	91
